# Supplementary material for: From sequence to enzyme mechanism using multi-label machine learning
Source: BMC Bioinformatics. 2014 May 19;15:150. doi: 10.1186/1471-2105-15-150 (PMC4229970; doi:10.1186/1471-2105-15-150)
Supplement: Additional file 2 — Java code of ml2db. Additional file ml2db_code.tar.gz contains the Java source code to run the multi-label machine learning experiments and save the results to database. The code’s Javadoc is included. [file 1471-2105-15-150-S2.zip › additional file 2/ml2db/utils/doc/edu/cornell/lassp/houle/RngPack/RandomSeedable.html]

RandomSeedable


JavaScript is disabled on your browser.


- Overview
- Package
- Class
- Use
- Tree
- Deprecated
- Index
- Help

- Prev Class
- Next Class

- Frames
- No Frames

- All Classes

- Summary:
- Nested |
- Field |
- Constr |
- Method

- Detail:
- Field |
- Constr |
- Method


edu.cornell.lassp.houle.RngPack

## Class RandomSeedable

- java.lang.Object
- - cern.colt.PersistentObject
  - - edu.cornell.lassp.houle.RngPack.RandomElement
    - - edu.cornell.lassp.houle.RngPack.RandomSeedable

- All Implemented Interfaces:
  :   java.io.Serializable, java.lang.Cloneable

  Direct Known Subclasses:
  :   RandomEngine, Ranecu, Ranlux, Ranmar

  ---

    

  ```
  public abstract class RandomSeedable
  extends RandomElement
  ```

  `RandomSeedable` is an abstract class that extends the
  `RandomElement` class to include the ability to automatically
  generate a valid `long` seed from the clock. Thus it provides a
  consistent interface for seeding interchangable generators. It is reccomended
  that a `RandomSeedable` have a constructor that takes a
  `long` for a seed. For example, if you write a generator called
  `ReallyRandom`, you want to be able to do

  ```
   long seed = ReallyRandom.ClockSeed();
   RandomSeedable e = new ReallyRandom(seed);
  ```

  this makes it convenient to keep a copy of the seed in case you want to
  restart the generator with the same seed some time in the future.

  If one is going to use a long to generate a smaller seed by taking
  `Clockseed()` modulus another number, we reccomend that you use a
  prime number; this ensures that the generator would have the maximum "period"
  if it were started at regular issues, for instance, by a batch job. See
  `Ranmar` for an example.

  Source
  code  is available.

  Version:
  :   1.0

  Author:
  :   Paul Houle  (E-mail:
      houle@msc.cornell.edu)

  See Also:
  :   `Ranecu`,
      `Ranlux`,
      `Ranmar`,
      Serialized Form

- - ### Constructor Summary

    Constructors

    Constructor and Description

    `RandomSeedable()`
  - ### Method Summary

    Methods

    | Modifier and Type | Method and Description |
    | --- | --- |
    | `static long` | `ClockSeed()` Return a long integer seed calculated from the date. |
    | `static long` | `ClockSeed(java.util.Date d)` Return a long integer seed given a date |

    - ### Methods inherited from class edu.cornell.lassp.houle.RngPack.RandomElement

      `choose, choose, gaussian, gaussian, powlaw, raw, raw, raw, uniform`
    - ### Methods inherited from class cern.colt.PersistentObject

      `clone`
    - ### Methods inherited from class java.lang.Object

      `equals, getClass, hashCode, notify, notifyAll, toString, wait, wait, wait`

- - ### Constructor Detail


    - #### RandomSeedable

      ```
      public RandomSeedable()
      ```
  - ### Method Detail


    - #### ClockSeed

      ```
      public static long ClockSeed()
      ```

      Return a long integer seed calculated from the date. Equivalent to
      `ClockSeed(new Date());`

      Returns:
      :   a long integer seed


    - #### ClockSeed

      ```
      public static long ClockSeed(java.util.Date d)
      ```

      Return a long integer seed given a date

      Parameters:
      :   `d` - a date

      Returns:
      :   a long integer seed


- Overview
- Package
- Class
- Use
- Tree
- Deprecated
- Index
- Help

- Prev Class
- Next Class

- Frames
- No Frames

- All Classes

- Summary:
- Nested |
- Field |
- Constr |
- Method

- Detail:
- Field |
- Constr |
- Method
